# Supplementary material for: The causal role of circulating inflammatory markers in osteoporosis: a bidirectional Mendelian randomized study
Source: Front Immunol. 2024 Jul 18;15:1412298. doi: 10.3389/fimmu.2024.1412298 (PMC11291241; doi:10.3389/fimmu.2024.1412298)
Supplement: Supplementary file 1 [file DataSheet_1.zip › supplementary material/OSTEOPOROSIS-CIM/GCST90274848.tsv.gz_forest.pdf]

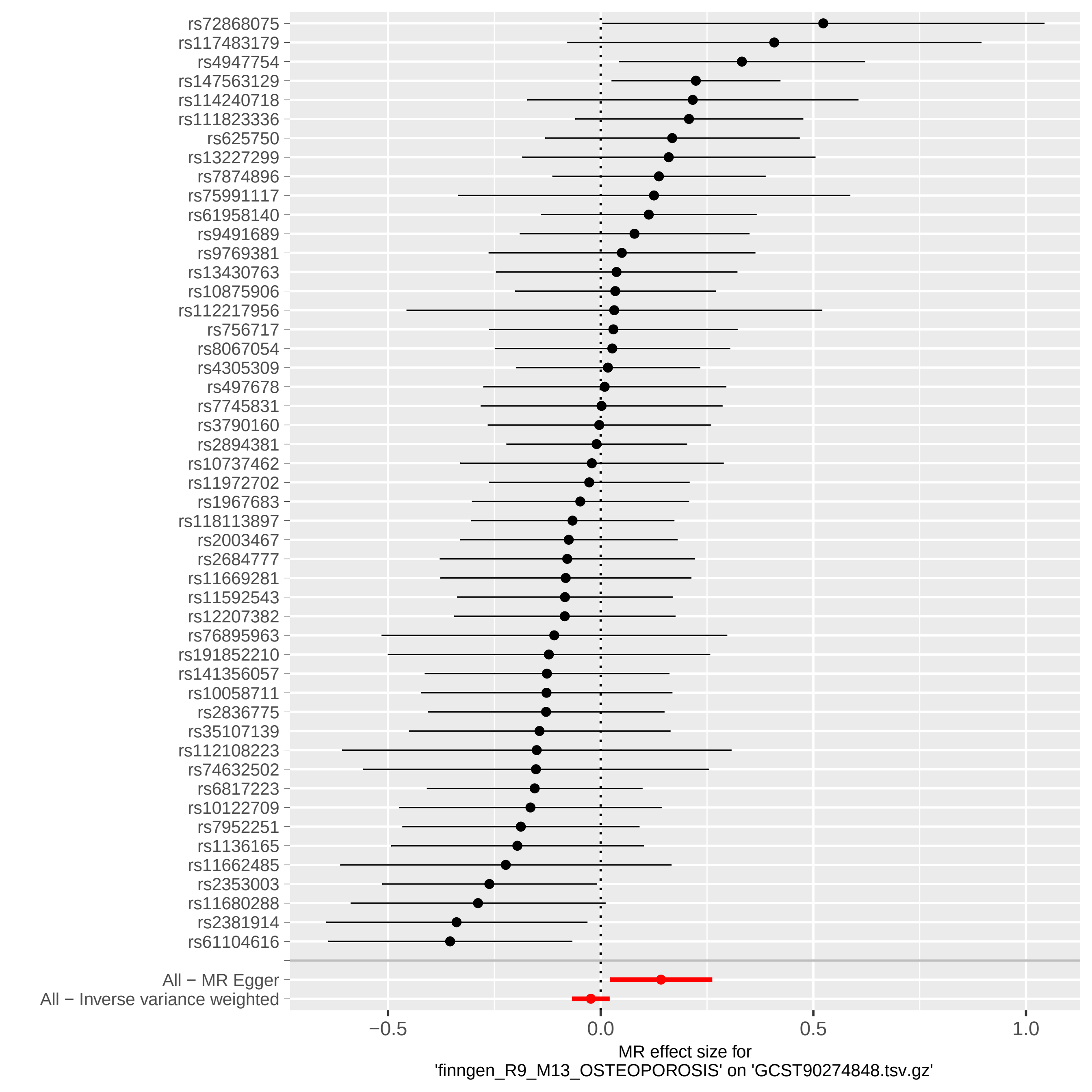

rs72868075
rs117483179
rs4947754
rs147563129
rs114240718
rs111823336
rs625750
rs13227299
rs7874896
rs75991117
rs61958140
rs9491689
rs9769381
rs13430763
rs10875906
rs112217956
rs756717
rs8067054
rs4305309
rs497678
rs7745831
rs3790160
rs2894381
rs10737462
rs11972702
rs1967683
rs118113897
rs2003467
rs2684777
rs11669281
rs11592543
rs12207382
rs76895963
rs191852210
rs141356057
rs10058711
rs2836775
rs35107139
rs112108223
rs74632502
rs6817223
rs10122709
rs7952251
rs1136165
rs11662485
rs2353003
rs11680288
rs2381914
rs61104616
All − MR Egger
All − Inverse variance weighted
−0.5
0.0
0.5
1.0
MR effect size for
'finngen_R9_M13_OSTEOPOROSIS' on 'GCST90274848.tsv.gz'
